# Milk: an exosomal microRNA transmitter promoting thymic regulatory T cell maturation preventing the development of atopy?

**DOI:** 10.1186/1479-5876-12-43

**Published:** 2014-02-12

**Authors:** Bodo C Melnik, Swen Malte John, Gerd Schmitz

**Affiliations:** 1 Department of Dermatology, Environmental Medicine and Health Theory, University of Osnabrück, Sedanstrasse 115, D-49090 Osnabrück, Germany; 2 Institute for Clinical Chemistry and Laboratory Medicine, University Hospital Regensburg, University of Regensburg, Josef-Strauss-Allee 11, D-93053 Regensburg, Germany

**Keywords:** Atopy prevention, DNA demethylation, Exosome, FoxP3, MicroRNA, Milk, MiR-155, Regulatory T cell

## Abstract

Epidemiological evidence confirmed that raw cow’s milk consumption in the first year of life protects against the development of atopic diseases and increases the number of regulatory T-cells (Tregs). However, milk’s atopy-protective mode of action remains elusive.

This review supported by translational research proposes that milk-derived microRNAs (miRs) may represent the missing candidates that promote long-term lineage commitment of Tregs downregulating IL-4/Th2-mediated atopic sensitization and effector immune responses. Milk transfers exosomal miRs including the ancient miR-155, which is important for the development of the immune system and controls pivotal target genes involved in the regulation of FoxP3 expression, IL-4 signaling, immunoglobulin class switching to IgE and FcϵRI expression. Boiling of milk abolishes milk’s exosomal miR-mediated bioactivity. Infant formula in comparison to human breast- or cow’s milk is deficient in bioactive exosomal miRs that may impair FoxP3 expression. The boost of milk-mediated miR may induce pivotal immunoregulatory and epigenetic modifications required for long-term thymic Treg lineage commitment explaining the atopy-protective effect of raw cow’s milk consumption.

The presented concept offers a new option for the prevention of atopic diseases by the addition of physiological amounts of miR-155-enriched exosomes to infant formula for mothers incapable of breastfeeding.

## Introduction

Children who grow up on traditional farms are protected from atopic diseases [1]. Early-life consumption of unboiled cow’s milk has been identified as the most protective factor for the development of atopy [2-10]. Farm milk exposure has been associated with increased numbers of CD4^+^CD25^+^FoxP3^+^ regulatory T cells (Tregs), lower atopic sensitization and asthma in 4.5-year-old children [11]. Treg cell numbers are negatively associated with asthma and perennial IgE levels [11]. However, potential effectors of milk, which stimulate the development of Tregs remain elusive. This review provides translational evidence that milk-derived exosomal microRNAs may be the potential stimuli for thymic Treg maturation and raw milk-mediated atopy prevention.

### Atopic diseases are associated with reduced Treg numbers

Atopic allergy is a Th2 cell-mediated disease that involves the formation of specific IgE antibodies against innocuous environmental substances. Both naturally occurring thymus-derived and inducible Tregs of the periphery prevent allergy development via suppression of Th2 cells [12-14]. Decreased FoxP3^+^ Treg numbers have been detected in atopic mothers at the 34^th^ week of gestation, in cord blood in association with high IgE levels, in sputum, nasal secretions and blood of atopic patients pointing to the pivotal role of FoxP3^+^ Tregs in the immunopathogenesis of atopy [15-18].

*Scurfy* is an X-linked recessive severe murine autoimmune disease resulting from a *Foxp3* mutant [18]. The human analog is the *immune dysregulation, polyendocrinopathy, enteropathy, X-linked (IPEX) syndrome* associated with eczema and increased IgE levels caused by impaired function of Tregs due to mutated *FOXP3*[19]. In the X-linked immunodeficiency disorder *Wiskott-Aldrich syndrome* (WAS) the mutated WAS protein (WASP) plays the key role in impaired Treg suppressor function [20,21]. WASP knockout mice display decreased numbers of Treg cells in both the thymus and peripheral lymphoid organs [22]. Tregs control the severity of anaphylaxis [23], contribute to the resolution of Der p1-induced allergic airway inflammation [24], and inhibit allergen-specific effector cells important for the successful outcome in allergen-specific immunotherapy [25]. Thus, Tregs are central players in the pathogenesis and treatment of atopy [26,27].

### Thymic maturation of natural regulatory T cells

**Figure 1:**
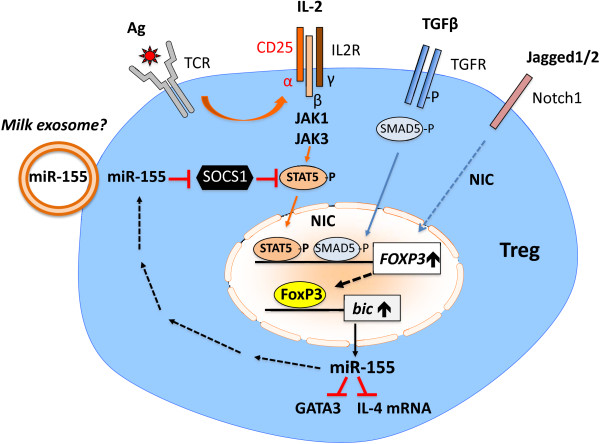
**Potential mechanisms of milk exosome miR-155-mediated FoxP3 expression in Tregs.** TCR activation upregulates CD25 and subsequent IL-2/STAT5 signaling, which cooperatively with TGFβ-activated SMAD5 stimulate the *FOXP3* promoter. MiR-155 attenuates the expression of SOCS1, the inhibitor of STAT5, thus amplifying IL-2/STAT5-mediated FoxP3 expression. FoxP3 activates the *bic* promoter enhancing the synthesis of miR-155, which suppresses mRNAs of GATA3 and IL-4, pivotal transcription factors of Th2-mediated IgE-driven atopic immune responses.

The vast majority of Tregs is generated in the thymic medulla at the CD4^+^ single-positive stage of thymocyte development [28,29]. Tregs highly express FoxP3, the master regulator for Treg cell differentiation and function [30-32]. Whereas naturally occurring Tregs are educated in the thymus, inducible Tregs can be generated in the periphery [26,33-41]. FoxP3^+^ T cells are detectable in the periphery 3 days after birth during the period of colostrum feeding [26]. Hassall’s corpuscles in the thymic medulla secrete *thymic stromal lymphopoietin* (TSLP) that activates CD11c^+^ dendritic cells (DCs), which induce Foxp3 expression in immature CD4^+^CD8^-^CD25^-^ thymocytes [42,43]. Crucial for FoxP3 induction are early signals from the T cell receptor (TCR), interleukin-2 (IL-2), transforming growth factor-β (TGF-β), and Notch1 [44,45] (Figure 1).

### Role of microRNAs in thymic FoxP3^+^ Treg cell maturation

MicroRNAs (miRs) are fundamental regulators of posttranscriptional programs that play a role in maturation and differentiation of Tregs in the thymus [46-49]. Substantial evidence underlines that miR-155 is required for the development of the Treg lineage [47]. MiR-155-deficient mice have reduced numbers of Tregs both in the thymus and periphery [47]. FoxP3, which is highly expressed in Tregs, binds to the promoter of *bic*, the gene encoding miR-155 [40,50,51]. TCR and Notch signaling upregulates the IL-2R α-chain (CD25), rendering thymocytes receptive to subsequent cytokine signals that foster their development into fully functional FoxP3^+^ Tregs [52-54]. IL-2 is capable of transducing signals in CD4^+^FoxP3^+^ Tregs as determined by STAT5 phosphorylation [54]. Deletion of miR-155 results in limited IL-2/STAT5 signaling and reduced Treg numbers [55]. Remarkably, miR-155 enhances FoxP3 expression by targeting suppressor of cytokine signaling 1 (SOCS1), an important negative regulator of IL-2R/STAT5 signaling (Figure 1). MiR-146a targets STAT1 and regulates Treg-mediated suppression function and maintains Treg identity [48]. Deletion of miR-146a in Tregs causes a severe autoimmune phenotype akin to *Dicer knockout animals*, characterized by increased numbers of poorly functional FoxP3^+^ Tregs in the periphery [56]. MiR-21 is highly expressed in Tregs and positively regulates *FOXP3*[57]. MiRs are processed in the cytoplasm by the ribonuclease Dicer. Conditional knockout of Dicer in CD4^+^ cells results in depletion of thymic Tregs and suppressed TGF-β-mediated induction of Foxp3 in naïve CD4^+^ cells associated with increased IL-4 levels [50]. Dicer knockout mice as well as mice with conditional knockout of Dicer in FoxP3^+^ cells develop severe autoimmune diseases [58,59]. In the later model, FoxP3 expression is unstable and Treg revert to an effector phenotype producing IL-4 and IFN-γ. Notably, miR-155 negatively regulates mRNA levels of GATA-3 and IL-4 [60]. It is well known that GATA-3 promotes Th2 responses [61]. In miR-155 null mice increased Th2 cell differentiation has been reported [62,63].

### Exosomal microRNAs in immune cell communication

Valadi et al. [64] were the first who demonstrated that exosome-mediated transfer of mRNAs and microRNAs is a novel mechanism of genetic exchange between cells. Secreted miRs represent a newly recognized layer of gene regulation and intercellular communication [65-67]. MiRs bind through partial sequence homology to the 3′-untranslated region of target mRNAs and cause either translational block or mRNA degradation [68]. Exosomal miRs, enclosed by membranous microvesicles, play a pivotal role for horizontal miR transfer [69]. Raposo *et al.*[70] provided first evidence for exosome-mediated immune cell communication. Unidirectional transfer of miR-loaded exosomes from T cells to antigen-presenting cells has recently been confirmed [71]. For immune cell-cell interactions exosome transport exchanging genetic messages over distances has been demonstrated [72,73]. In the human thymic medulla miR-transporting exosomes that may provide genetic signals required for Treg formation have recently been characterized [74].

### Milk-derived exosomal miRs: boosters for thymic Treg maturation?

Recently, we have suggested that milk is an endocrine signaling system that promotes mTORC1 signaling by transfer of essential branched-chain amino acids and exosomal regulatory miRs to the milk recipient [75]. Zhang et al. [76] published that diet-derived plant MIR168a reaches the plasma compartment of human subjects and affects LDLRAP1 metabolism in the liver [76]. However, Dickinson et al. [77] were unable to detect plant miRs after feeding in mice. Breast milk in comparison to all other body fluids contains the highest amounts of total RNAs [78]. Bovine and human milk contain substantial amounts of exosomal miRs that may be transferred to the infant to promote immune regulatory functions [79-81]. MiR-containing exosomes of 30-100 nm diameter have been identified in human breast milk, cow’s milk, bovine whey and colostrum [83-85]. Exosomes from bovine colostrum and mature milk are able to deliver miRs into cultured cells thereby increasing cytoplasmic miR levels [85]. Although not proven yet, several investigators regard milk-derived miRs as important effectors for the development of the infant’s immune system and proposed that milk’s miRs may reach the infant’s circulation and organ systems [81,85-87]. Admyre *et al.*[82] demonstrated that incubation of human PBMCs with isolated human milk exosomes increased the number of CD4^+^CD25^+^FoxP3^+^ Tregs in a dose-dependent manner. Human and bovine milk contain significant amounts of those immune regulatory miRs (miR-155, miR-146a, miR-21) that play a known role in thymic Treg differentiation [80,81,85,87]. Bovine colostrum in comparison to mature milk contains the highest amounts of miR-155 and miR-21 [80,81]. Substantial exosomal miR-155 content has been detected in bovine whey [81]. The lipid bilayer of milk exosomes protects their miR-cargo against harsh degrading conditions like low acidic pH of 1-2 and RNase-mediated degradation [81,86]. Boiling of milk, however, results in complete miR degradation [87]. Raw cow’s milk contains the highest amounts of bioactive miRs, whereas pasteurized milk contains lower levels and milk powder used for infant formula production only exhibits trace amounts of detectable RNAs [80,81].

### Milk exosome CD81: an exosomal antigen required for thymocyte maturation?

The increased intestinal permeability during the postnatal period may support milk exosome traffic into the infant’s blood circulation. Intestinal cells release exosomes of 30-90 nm in diameter from their apical and basolateral sides [87]. Milk exosomes are specifically characterized for the presence of CD81, CD63 and Hsc70 and the absence of calnexin [83]. The tetraspanins CD81 and CD63 are also present on intestinal cell-derived exosomes [88] and circulating exosomes in human plasma [89]. Blood is regarded as a physiological fluid for exosome circulation in the body supporting exosome traffic for cell-cell and organ-organ communications [67,71-73,90]. Importantly, exosomes have been observed in murine and human thymus [74,91]. Milk exosomes and thymic exosomes are of comparable size and contain TSG101, CD81, CD63 and milk fat globulin (MFG)-8 [74,83,91-93]. A monoclonal antibody against CD81 blocked the appearance of αβ T cells in fetal murine thymic organ cultures. In reaggregation cultures with CD81-transfected fibroblasts, CD4^-^CD8^-^ thymocytes differentiated into CD4^+^CD8^+^ T cells. Thus, interaction between immature thymocytes and CD81 is required for the transition of thymocytes from the CD4^-^CD8^-^ to the CD4^+^CD8^+^ stage [93]. CD81 of thymic stromal cells but also milk exosome-derived CD81 may play a role in CD81-mediated thymocyte development.

### Milk- and thymus-derived exosomes promote Treg maturation

MiR-155 exhibits highest expression in colostrum and is still a predominant exosomal miR of bovine whey [84,85]. MiR-155, miR-146a and miR-21 are components of human plasma [86,94,95]. Human and bovine milk exosomes are taken up by macrophages and thereafter increase cellular miR levels [85,92]. Notably, isolated human breast milk exosomes incubated with human PBMCs increase CD4^+^CD25^+^FoxP3^+^ Treg numbers [82]. Intriguingly, murine thymic exosomes induce the conversion of CD4^+^CD25^-^ thymic T cells into CD4^+^CD25^+^FoxP3^+^ Tregs in a dose-dependent manner [91]. Thus, both human breast milk exosomes and thymic exosomes are able to induce CD4^+^CD25^+^FoxP3^+^ Tregs [82,91]. We thus propose that milk may function as a miR messenger system boosting thymic Treg cell maturation by transfer of milk-derived exosomes donating miRs required for appropriate thymic Treg maturation by the evolutionarily conserved process of breastfeeding. Notably, phylogenetic studies demonstrate that miR-155 is conserved across species [96]. There is a high homology between human (hsa-mir-155) and bovine miR-155 (bta-mir-155) (http://www.mirbase.org).

### MiR-mediated *FOXP3* demethylation

**Figure 2:**
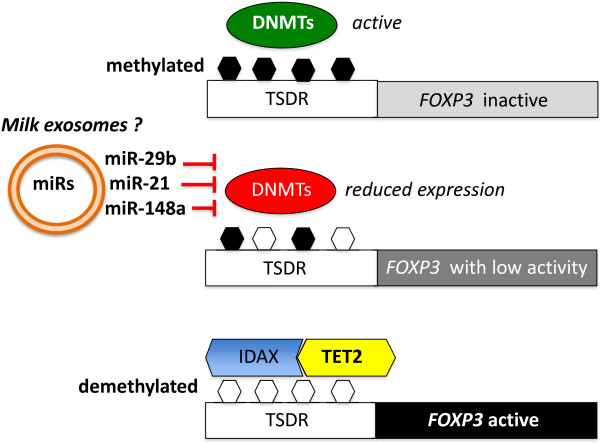
**Proposed mode of action of milk miR-mediated demethylation of *FOXP3*.** Milk-derived miR-29b, -21, and -148a may reduce the expression of DNMTs resulting in TSDR hypomethylation required for IDAX binding, which finally attracts TET2 to the TSDR resulting in complete TSDR demethylation, important for permanent *FOXP3* stabilization.

Farm milk exposure increases the numbers of demethylated CD4^+^CD25^+^FoxP3^+^ Tregs [11]. Stable expression of Foxp3 in Tregs depends on DNA demethylation at the *Treg-specific demethylated region* (TSDR), a conserved, CpG-rich region within the *FOXP3* locus [97]. Binding of the transcription factor Ets-1 to the demethylated Foxp3 gene stabilizes Foxp3 expression in Tregs [98] (Figure 2). Atopic individuals express lower numbers of demethylated FoxP3^+^ Tregs [99]. DNA methylation is often associated with inhibition of transcriptional activity and plays a fundamental role during development and genomic imprinting [100]. Milk, the “starter cocktail” of postnatal mammalian life, may function as an epigenetic regulator for thymic Treg maturation. There are two potential mechanisms of DNA demethylation: 1) passive demethylation through inhibition of DNA methyltransferases (DNMTs) and 2) active demethylation mediated by ten-eleven-translocation (TET) 2 and 3 [100]. TET2 binding to CpG-rich regions requires the interaction of TET2 with the protein IDAX (also known as CXXC4) [101]. Intriguingly, the CXXC DNA-binding domains can bind unmethylated DNA and recruit TET2 via IDAX [102]. Both DNMT1 and DNMT3b are associated with the *Foxp3* locus in CD4^+^ cells [103]. Methylation of CpG residues represses Foxp3 expression, whereas complete demethylation is required for stable Foxp3 expression [103]. There is increasing evidence that miRs modify the regulatory network of TET2 expression [104]. MiR-21 indirectly down-regulates DNMT1 by targeting *ras guanyl nucleotide-releasing protein 1* (RASGRP1). MiR-148a, a highly expressed miR-species in colostrum and mature milk [80,81,87], directly targets DNMT1 expression [105]. MiR-29b, another miR species of milk [81], contributes to DNA hypomethylation of CD4^+^ T cells in systemic lupus erythematosus indirectly targeting DNMT1 [106]. Milk-miR-mediated hypomethylation of CpG-regions of the TSDR *FOXP3* locus may thus promote the final step of active TSDR demethylation. In fact, IDAX-mediated TET2 binding results in complete and permanent *FOXP3* demethylation. TSDR demethylation occurs during the CD4-single positive stage of thymocytes and the presence of 5-hydroxymethylcytosine (5-hmC), a product of TET-mediated 5mC hydroxylation, within the TSDR region and the induction of TET2/3 during Treg maturation points to active TET-mediated demethylation of *FOXP3* TSDR [97]. We thus speculate that the immunoregulative miR network of milk may induce lineage-specific epigenetic modifications of *FOXP3* required for long-term Treg lineage stability and atopy prevention.

### MiR-155 and atopy-related target genes

MiR-155 inhibits *suppressor of cytokine signaling 1* (SOCS1) [60]. SOCS1 is a negative regulator of phosphorylated STAT5. TCR, IL-2 and TGF-β1 are pivotal signals for Treg differentiation associated with phosphorylation of STAT5 and SMAD5, which enhance FoxP3 expression. MiR-155-mediated suppression of SOCS1 augments FoxP3 expression promoting further miR-155 synthesis [47] (Figure 1).

**Figure 3:**
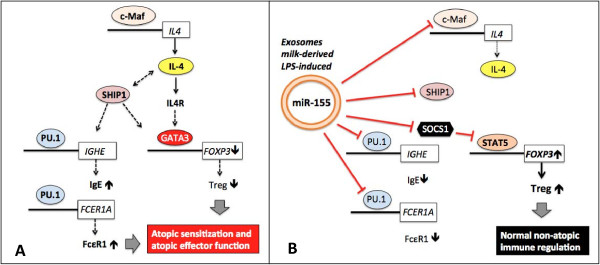
**Impact of miR-155 on immune regulating transcription factors. A)** atopic immune deviations in the absence of miR-155 and **B)** non-atopic development of the immune system due to appropriate miR-155 signaling.

The transcription factor c-Maf promotes IL-4 expression and is induced during normal precursor cell differentiation along the Th2 lineage [107,108]. c-Maf binds to the c-Maf response element in the proximal IL-4 promoter (Figure 3A). Importantly, c-Maf is a target of miR-155 [62,109]. In accordance, CD4^+^ cells transfected with anti-miR-155 expressed higher levels of GATA-3 and IL-4 [60]. Thus, miR-155-mediated c-Maf suppression promotes FoxP3^+^ Treg activity and impairs IL-4/Th2 cell-mediated atopic immune deviations (Figure 3B).

The lipid phosphatase SHIP1 (*Src homology-2 domain-containing inositol 5-phosphatase 1*) plays an increasing role for immune regulation [110,111]. Myeloid-specific ablation of SHIP leads to the expansion of Treg cell numbers, confirming the role of SHIP in the control of Treg numbers [112]. Notably, SHIP1 mRNA is a primary target miR-155 [113].

The transcription factor PU.1 is a direct target of miR-155 and is involved in the regulation of immunoglobulin (Ig) class-switch of plasma cells [114]. Ig heavy chain class switching to IgE is directed by IL-4 and IL-13 by inducing transcription from the IgE germline promoter [115]. IL-4-induced IgE germline gene transcription represents an early step during IgE isotype switch differentiation and is orchestrated by the coordinated action of the transcription factors STAT6, PU.1, NF-κB, and C/EBP on the promoter region of the IgE germline gene [115-117]. MiR-155-mediated suppression of PU.1 and c-Maf may thus attenuate IL-4-induced IgE synthesis. Notably, PU.1 cooperatively with GATA-1 transactivates the α-chain of the high affinity receptor of IgE (FcϵRI) [118]. FcϵRI plays an important role in IgE-mediated atopic sensitization as well as in IgE-mediated atopic immune reactions.

Cytotoxic T lymphocyte-associated antigen-4 (CTLA-4) is a surface molecule of activated T cells and a negative regulator of T-cell activation. The mean percentage of T cells expressing CTLA-4 in patients with atopic dermatitis was higher than in the control group [119]. miR-155 was identified as a direct target of CTLA-4 [120]. CTLA-4 has been shown in mice to control Foxp3+ regulatory T cell function [121].

### Increased maternal LPS-exposure during farming enhances miR-155 release

Pregnancy in a farm environment reduces the infant’s risk of atopic diseases [1-9]. TLR-mediated innate response pathways are believed to attenuate allergic Th2-driven immune responses [122]. Blood cells of infants of farming-exposed mothers exhibit upregulated expression of TLR2 and TLR4 [123,124]. MiRs are fine-tuners of TLR signaling and play a crucial role in endotoxin tolerance [125,126]. Enhanced exposure of lipopolysaccharides (LPS) in the farm environment may result in stronger LPS-mediated TLR4 activation in monocyte/macrophages and DCs that increase expression of miR-155, miR-146a, and miR-21, crucial regulatory miRs involved in thymic Treg maturation [127-133]. Notably, activated macrophages release exosomes that can activate and recruit immune cells [134].

Placental trophoblasts, which form the interface between the maternal environment and the fetus, on stimulation secrete miR-loaded exosomes [135]. Low dose injection of LPS induces miR-155 and pre-eclampsia-like symptoms in the rat and elevated placental miR-155 in pre-eclampsia patients [136]. A time-and dose-dependent accumulation of miR-155 following LPS stimulation has been observed in human trophoblast cells [136]. LPS-stimulated placenta-derived exosomal miR-release may modify fetal immune regulation [137]. Thus, enhanced prenatal LPS-induction of miR-155 may explain the higher Treg numbers in umbilicial cord blood of newborn infants of farming exposed mothers [138]. LPS-mediated miR-155-expression with subsequent SOCS1 inhibition could result in robust TLR4/JAK-STAT signaling further amplifying LPS-induced miR-155 responses [139,140].

### Bioactive exosomal miR-155 in raw cow’s milk

Feeding raw, unpasteurized cow´s milk in the first year of life exerts atopy-preventive effects, increases the number and function of FoxP3^+^ Tregs and decreases IgE plasma levels [1-9]. Boiling of milk degrades bioactive miRs in cow’s milk [87]. Especially the whey protein fraction of milk has been implicated to mediate the atopy-protective effect of raw farm milk [3,8]. Noteworthy, miR-155 and miR-146a have not been detected in the lipid fraction of human breast milk [141]. MiR-155 is expressed in highest amounts in colostrum and is a substantial component of the whey fraction of mature bovine milk [80,81,85]. Exosome membrane integrity is essential for the uptake of milk miRs into cultured cells [85]. The boiling process may disrupt the lipid bilayer of milk exosomes exposing their miR content to RNase-mediated degradation.

### Breastfeeding of non-atopic *versus* atopic mothers and Treg maturation

A history of breastfeeding is associated with a reduction of the risk of asthma and atopic dermatitis [142]. Apparently, after termination of placenta-mediated signaling towards the thymus, the immunoregulatory program featured by the mammary gland may tune final miR-dependent events for the proper development of the infant’s immune system. We speculate that atopic mothers who exhibit lower numbers of FoxP3^+^ Tregs and who may accordingly express lower levels of FoxP3-stimulated miR-155 may provide deficient amounts of breast milk miR-155 to their infants. This may explain why atopic mothers transmit atopic diseases more frequently than atopic fathers [143-145].

### Artificial formula feeding and Treg maturation

Breastfeeding compared to artificial formula feeding exerts atopy-preventive effects [142,146,147]. Formula production is based on bovine milk protein powder, which in comparison to raw cow’s milk only contains minimal amounts of RNA [81]. Extensively hydrolyzed formula made for “atopy prevention” exhibits the lowest miR levels compared to standard formula [81,148]. According to the *American Academy of Pediatrics Committee on Nutrition and Section on Allergy and Immunology*, there is only “modest evidence” that the onset of atopic disease may be delayed or prevented by feeding hydrolyzed formulas compared with standard formula [146].

## Conclusion

Exosomal cargo transfer plays an increasing role for intercellular communication [149,150]. Accumulating translational evidence sheds a new light on the potential role of milk as a transmitter of exosome-derived immune regulatory miRs for thymic Treg maturation. Milk miRs may promote the two-step selection process turning self-reactive thymocytes into stable Treg cells. TCR stimulated IL-2/STAT5 signaling may be enhanced by milk miR-155-mediated SOCS1 suppression, which augments upregulation of FoxP3. FoxP3 promotes miR-155 expression further enhancing this feed forward regulatory circuit. In a second step FoxP3 expression may be stabilized by milk-miR-mediated hypomethylation of the TSDR region of *FOXP3*. This hypomethylation may allow IDAX binding, which finally attracts TET2 promoting active demethylation stabilizing FoxP3 maturation and long-term Treg lineage commitment.

**Figure 4:**
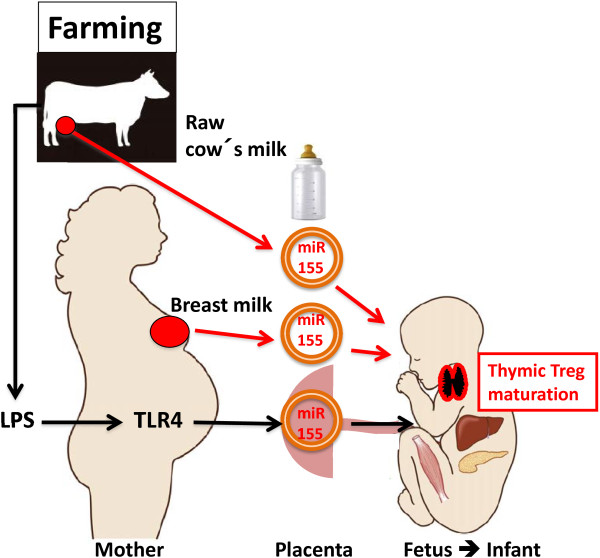
**Perinatal scenario of milk-and LPS-induced miR-155-exosome-signaling promoting thymic Treg maturation.** Fetuses and infants raised in an active farming environment are exposed to abundant sources of LPS and raw cow’s milk that either stimulate or provide miR-155 compared to infants raised under civic conditions and artificial formula feeding.

Furthermore, functionally active FoxP3 Treg cells suppress the development of Th2 cell-dependent immune responses. Milk miR-155 may impair atopic sensitization by suppression of c-Maf/SHIP1-mediated IL-4 synthesis, PU.1-mediated Ig class switch to IgE as well as PU.1-driven FcϵRI α-chain synthesis. Thus, the *milk miR system* may not only augment thymic Treg maturation but may apparently prevent Th2-mediated atopic sensitization and atopic effector responses. Boiling of milk obviously destroys the miR-signaling system of milk, whereas LPS-mediated miR-155 release from stimulated maternal macrophages and trophoblast cells as well as fresh cow’s milk-mediated miR-155 transfer may promote thymic Tregs maturation explaining synergistic atopy-preventive effects of perinatal farm exposure (Figure 4). Milk appears to function as an evolutionarily highly conserved miR-dependent epigenetic modifyer imprinting appropriate changes required for long-term thymic FoxP3-mediated Treg differentiation. Milk-derived exosomes in synergy with thymic exosomes may play the essential role for stable maturation of CD4^+^CD25^+^FoxP3^+^ Tregs, which themselves following TCR activation produce CD73-containing exosome-like structures that mediate their suppressive activity [151].

Obviously, atopic individuals are “Treg weaklings” exhibiting lower numbers and function of FoxP3 Tregs compared to non-atopic subjects. Breast milk of atopic mothers may thus provide less FoxP3-induced miR-155 explaining the increased maternal transmission of atopic diseases compared to the lower paternal atopy transmission. Accumulating evidence supports our concept that milk’s exosomal miR system may represent “the missing candidate” inducing the atopy-preventive effects of raw cow’s milk consumption (Table 1). Deviations of miR processing and miR-regulated transcriptional activity may play a future role for a deeper understanding of the immunopathogenesis and treatment of atopic diseases. Future prevention of atopic diseases might be possible by addition of appropriate miR-155-enriched exosomes to artificial infant formula.

**Table 1:** Translational evidence for milk-microRNA-mediated thymic Treg maturation

**Potential function of milk microRNA**	**Comment**	**References**
Milk contains abundant miRs	From all body fluids human milk contains the highest amounts of RNAs and miRs	[78-82,84-87,92]
Milk contains miR-155, miR-146a, and miR-21	MiR-155, miR-146a and miR-21 are crucial miRs involved in Treg maturation and function	[79-81,84,85,87]
The majority of milk’s miRs are transported in exosomes	Exosomes transfer genetic information for cell-cell communications over short and long distances	[81,82,84-87,92]
MiR-155 is a component of colostrum and bovine whey and is found to be transported in exosomes	MiR-155 is an ancient highly conserved miR involved in immune regulation	[84,85]
Milk exosomes are resistant against RNase-degradation and acidic conditions (pH1-2)	Milk exosomes may survive the acidic environment of the stomach. Boiling of milk destroys the biological activity of milk miRs	[80,81,86]
Mir-155, miR-146a and miR-21 are components of human blood plasma	Milk miR-containing exosomes may be transported in circulation and may reach the thymus	[94,95]
Bovine colostrum and bovine milk and human breast milk exosomes containing miRs are taken up by cells and increase cytoplasmic miR levels	Milk-derived miRs may be taken up by exosome endocytosis in recipient cells. Physical destruction of exosomal lipid bilayer structure abolishes cellular miR uptake	[82,85,92]
Exosomal transfer is a known mechanism of communication between immune cells	Macrophages, B-cell, T cells and thymocytes communicate via exosome transfer	[70-73,91]
Human breast milk exosomes when added to PBMCs induce FoxP3^+^ Tregs	Breast milk miR-155 may induce the expression of FoxP3^+^ by inhibiting SOCS1 signaling	[82]
Exosomes have been detected in the murine and human thymus	Milk-derived exosomes may augment Treg cell maturation in the thymus	[74,91]
Murine thymic exosomes when added to thymus CD4^+^CD25^-^ T cells induce CD4^+^CD25^+^FoxP3^+^ Treg cells	Milk-derived exosomes may promote Treg cell formation of developing thymocytes within the human thymic medulla	[74,91]
MiR-21 and miR-29b inhibit DNMT1 expression in T cells	Milk miR-21 and miR-29b may promote stable expression of demethylated FoxP3 and thus lineage commitment of thymic Treg cells	[105,106]

## Abbreviations

CD63: Melanoma-associated antigen MLA1; CD73: Ecto-5-nucleotide enzyme; CD81: Target of antiproliferative antibody (TAPA1); DC: Dentritic cell; Der p1: Dermatophagoides pteronyssinus; DNMT: DNA methyltransferase; FcϵRI: IgE high affinity receptor; FCER1A: Fc fragment of IgE, high affinity I, receptor for, alpha subunit; FoxP3: Forkhead box P3 (scurfin); 5-hmc: 5-Hydroxymethylcytosine (5-hmC); IDAX: Inhibitor of DVL/axin complex (CXXC4); Ig: Immunoglobulin; IgE: Immunoglobulin E; IGHE: Immunoglobulin heavy epsilon chain; IL: Interleukin; IPEX: Immune dysregulation, polyendocrinopathy, enteropathy, X-linked syndrome; LC: Langerhans cell; LDLRAP1: Low density lipoprotein receptor adaptor protein 1; LPS: Lipopolysaccharide; MFG-8: Milk fat globulin-8; 5mC: 5-Methylcytosine; miR: Micro ribonucleic acid; NIC: Notch intracellular domain; PBMC: Peripheral blood mononuclear cell; SHIP1: Src homology-2 domain-containing inositol 5-phosphatase 1; SMAD: Mothers against decapentaplegic; SOCS1: Suppressor of cytokine signaling 1; STAT: Signal transducer and activator of transcription; TCR: T cell receptor; TET: Ten-eleven-translocation; TGF: Transforming growth factor; TLR: Toll-like receptor; Treg: Regulatory T cell; TSDR: Treg-specific demethylated region; TSG101: Tumor susceptibility gene 101; TSLP: Thymic stromal lymphopoietin; WAS: Wiskott-Aldrich syndrome; WASP: WAS protein.

## Competing interests

The authors declare that they have no competing interests.

## Authors’ contributions

BCM performed translational research and wrote the manuscript. SMJ proved the data on atopy epidemiology and GS analyzed the data on exosome microRNA biology. All authors read and approved the final manuscript.

## References

[B1] Von Mutius E Vercelli D Farm living: effects on childhood asthma and allergy Nat Rev Immunol 2010 10 861 868 21060319 10.1038/nri2871

[B2] Perkin MR Strachan DP Which aspects of the farming lifestyle explain the inverse association with childhood allergy? J Allergy Clin Immunol 2006 117 1374 1381 16751000 10.1016/j.jaci.2006.03.008

[B3] Loss G Apprich S Waser M Kneifel W Genuneit J Büchele G The protective effect of farm milk consumption on childhood asthma and atopy: the GABRIELA study J Allergy Clin Immmunol 2011 128 766 773 10.1016/j.jaci.2011.07.048 21875744

[B4] Braun-Fahrländer C von Mutius E Can farm milk consumption prevent allergic diseases? Clin Exp Allergy 2011 41 29 35 21155907 10.1111/j.1365-2222.2010.03665.x

[B5] Illi S Depner M Genuneit J Horak E Loss G Strunz-Lehner C Protection from childhood asthma and allergy in Alpine farm environments – the GABRIEL Advanced Studies J Allergy Clin Immunol 2012 129 1470 1477 22534534 10.1016/j.jaci.2012.03.013

[B6] Loss G Bitter S Wohlgensinger J Frei R Roduit C Genuneit J Prenatal and early-life exposures alter expression of innate immunity genes: the PASTURE cohort study J Allergy Clin Immunol 2012 130 523 530 22846753 10.1016/j.jaci.2012.05.049

[B7] von Mutius E Maternal farm exposure/ingestion of unpasteurized cow’s milk and allergic disease Current Opin Gastroenterol 2012 28 570 576 10.1097/MOG.0b013e32835955d3 23041676

[B8] Wlasiuk G Vercelli D The farm effect, or: when, what and how a farming environment protects from asthma and allergic disease Curr Opin Allergy Clin Immunol 2012 12 461 466 22892709 10.1097/ACI.0b013e328357a3bc

[B9] Lluis A Schaub B Lessons from the farm environment Curr Opin Allergy Clin Immunol 2012 12 158 163 22306551 10.1097/ACI.0b013e32835109a8

[B10] Sozanska B Pearce N Dudek K Cullinan P Consumption of unpasteurized milk and its effects on atopy and asthma in children and adult inhabitants in rural Poland Allergy 2013 68 644 650 23534445 10.1111/all.12147

[B11] Lluis A Depner M Gaugler B Saas P Casaca VI Raedler D Increased regulatory T-cell numbers are associated with farm milk exposure and lower atopic sensitization and asthma in childhood J Allergy Clin Immunol 2013 August 28 [Epub ahead of print] 10.1016/j.jaci.2013.06.034 23993223

[B12] Palomares O Yaman G Azkur A Akkoc T Akdis M Akdis CA Role of Treg in immune regulation of allergic diseases Eur J Immunol 2010 40 1232 1240 20148422 10.1002/eji.200940045

[B13] Ostroukhova M Ray A CD25+ T cells and regulation of allergen-induced responses Curr Allergy Asthma Rep 2005 5 35 41 15659261 10.1007/s11882-005-0052-6

[B14] Fujita H Meyer N Akdis M Akdis CA Mechanisms of immune tolerance to allergens Chem Immunol Allergy 2012 96 30 38 22433368 10.1159/000331868

[B15] Kawayama T Matsunaga K Kaku Y Yamaguchi K Kinoshita T O’Byrne PM Decreased CTLA4(+) and FoxP3(+)CD25(high)CD4(+) cells in induced sputum from patients with mild atopic asthma Allergol Int 2013 62 203 213 23524650 10.2332/allergolint.12-OA-0492

[B16] Stelmaszczyk-Emmel A Zawadzka-Krajewska A Szypowska A Kulus M Demkow U Frequency and activation of CD4 + CD25 FoxP3+ regulatory T cells in peripheral blood from children with atopic allergy Int Arch Allergy Immunol 2013 162 16 24 23817221 10.1159/000350769

[B17] Hinz D Simon JC Maier-Simon C Milkova L Röder S Sack U Reduced maternal regulatory T cell numbers and increased T helper type 2 cytokine production are associated with elevated levels of immunoglobulin E in cord blood Clin Exp Allergy 2010 40 419 426 20067477 10.1111/j.1365-2222.2009.03434.x

[B18] Brunkow ME Jeffery EW Hjerrild KA Paeper B Clark LB Yasayko SA Disruption of a new forkhead/winged-helix protein, scurfin, results in the fatal lymphoproliferative disorder of the scurfy mouse Nat Genet 2001 27 68 73 11138001 10.1038/83784

[B19] Barzaghi F Passerini L Bacchetta R Immune dysregulation, polyendocrinopathy, enteropathy, X-linked syndrome: a paradigm of immunodeficiency with autoimmunity Front Immunol 2012 3 211 23060872 10.3389/fimmu.2012.00211 PMC3459184

[B20] Humblet-Baron S Sather B Anover S Becker-Herman S Kasprowicz DJ Khim S Wiskott-Aldrich syndrome protein is required for regulatory T cell homeostasis J Clin Invest 2007 117 407 418 17218989 10.1172/JCI29539 PMC1764857

[B21] Marangoni F Trifari S Scaramuzza S Panaroni C Martino S Notarangelo LD WASP regulates suppressor activity of human and murine CD4(+)CD25(+)FoxP3(+) natural regulator T cells J Exp Med 2007 204 369 380 17296785 10.1084/jem.20061334 PMC2118740

[B22] Maillard MH Cotta-de-Almeida V Takeshima F Nguyen DD Michetti P Nagler C The Wiskott-Aldrich syndrome protein is required for the function of CD4(+)CD25(+)FoxP3(+) regulatory T cells J Exp Med 2007 204 381 391 17296786 10.1084/jem.20061338 PMC2118715

[B23] Kanjarawi R Dy M Bardel E Sparwasser T Dubois B Mecheri S Regulatory CD4 + FoxP3+ T cells control the severity of anaphylaxis PLoS One 2013 8 e69183 23922690 10.1371/journal.pone.0069183 PMC3724852

[B24] Leech MD Benson RA De Vries A Fitch PM Howie SE Resolution of Der p1-induced allergic airway inflammation is dependent on CD4 + CD25 + FoxP3+ regulatory cells J Immunol 2007 179 7050 7058 17982096 10.4049/jimmunol.179.10.7050

[B25] Akdis CA Akdis M Mechanisms and treatment of allergic disease in the big picture of regulatory T cells J Allergy Clin Immunol 2009 123 735 746 19348912 10.1016/j.jaci.2009.02.030

[B26] Sakaguchi S Miyara M Costantino CM Hafler DA FOXP3+ regulatory T cells in the human immune system Nat Rev Immunol 2010 10 490 500 20559327 10.1038/nri2785

[B27] Paik Y Dahl M Fang D Calhoun K Update: the role of FoxO3 in allergic disease Curr Opin Otolaryngol Head Neck Surg 2008 16 275 279 18475085 10.1097/MOO.0b013e3282ffabdc

[B28] Hsieh CS Lee HM Lio WC Selection of regulatory T cells in the thymus Nat Rev Immunol 2012 12 157 167 22322317 10.1038/nri3155

[B29] Fontenot JD Rasmussen JP Williams LM Dooley JL Farr AG Rudensky AY Regulatory T cell lineage specification by the forkhead transcription factor foxp3 Immunity 2005 22 329 341 15780990 10.1016/j.immuni.2005.01.016

[B30] Buckner JH Ziegler SF Functional analysis of FOXP3 Ann N Y Acad Sci 2008 1143 151 169 19076349 10.1196/annals.1443.014

[B31] Hori S Nomura T Sakaguchi S Control of regulatory T cell development by the transcription factor Foxp3 Science 2003 299 1057 1061 28115586

[B32] Yagi H Nomura T Nakamura K Yamazaki S Kitawaki T Hori S Crucial role of *FOXP3* in the development and function of human CD25 + CD4+ regulatory T cells Int Immunol 2004 16 1643 1656 15466453 10.1093/intimm/dxh165

[B33] Ziegler SF FOXP3: of mice and men Annu Rev Immunol 2006 24 209 226 16551248 10.1146/annurev.immunol.24.021605.090547

[B34] Lee HM Bautista JL Hsieh CS Thymic and peripheral differentiation of regulatory T cells Adv Immunol 2011 112 25 71 22118406 10.1016/B978-0-12-387827-4.00002-4

[B35] Bilate AM Lafaille JJ Induced CD4 + Foxp3+ regulatory T cells in immune tolerance Annu Rev Immunol 2012 30 733 758 22224762 10.1146/annurev-immunol-020711-075043

[B36] Baecher-Allan C Viglietta V Hafler DA Human CD4 + CD25+ regulatory T cells Semin Immunol 2004 16 89 98 15036232 10.1016/j.smim.2003.12.005

[B37] Hori S Sakaguchi S Foxp3: a critical regulator of the development and function of regulatory T cells Microbes Infect 2004 6 745 751 15207821 10.1016/j.micinf.2004.02.020

[B38] Zhang L Zhao Y The regulation of Foxp3 expression in regulatory CD4(+)CD25(+)Tcells: multiple pathways on the road J Cell Physiol 2007 211 590 597 17311282 10.1002/jcp.21001

[B39] Campbell DJ Ziegler SF FOXP3 modifies the phenotypic and functional properties of regulatory T cells Nat Rev Immunol 2007 7 305 310 17380159 10.1038/nri2061

[B40] Marson A Kretschmer K Frampton GM Jacobsen ES Polansky JK MacIsaac KD Foxp3 occupancy and regulation of key target genes during T-cell stimulation Nature 2007 445 931 935 17237765 10.1038/nature05478 PMC3008159

[B41] Fontenot JD Dooley JL Farr AG Rudensky AY Developmental regulation of Foxp3 expression during ontogeny J Exp Med 2005 202 901 906 16203863 10.1084/jem.20050784 PMC2213175

[B42] Watanabe N Wang YH Lee HK Ito T Wang YH Cao W Hassall’s corpuscles instruct dendritic cells to induce CD4 + CD25+ regulatory T cells in human thymus Nature 2005 436 1181 1185 16121185 10.1038/nature03886

[B43] Jiang Q Su H Knudsen G Helms W Su L Delayed functional maturation of natural regulatory T cells in the medulla of postnatal thymus: role of TSLP BMC Immunol 2006 7 6 16579866 10.1186/1471-2172-7-6 PMC1450317

[B44] Fontenot JD Gavin MA Rudensky AY FoxP3 programs the development and function of CD4 + CD25+ regulatory T cells Nat Immunol 2003 4 330 336 12612578 10.1038/ni904

[B45] Del Papa B Sportoletti P Cecchini D Rosati E Balucani C Balsdoni S Notch1 modulates mesenchymal stem cells mediated regulatory T-cell induction Eur J Immunol 2013 43 182 187 23161436 10.1002/eji.201242643

[B46] Xiao C Rajewsky K MicroRNA control in the immune system: basic principles Cell 2009 136 26 36 19135886 10.1016/j.cell.2008.12.027

[B47] Kohlhaas S Garden OA Scudamore C Turner M Okkenhaug K Vigorito E Cutting edge: the FoxP3 target miR-155 contributes to the development of regulatory T cells J Immunol 2009 182 2578 2582 19234151 10.4049/jimmunol.0803162

[B48] Josefowicz SZ Lu LF Rudensky AY Regulatory T cells: menchanisms of differentiation and function Annu Rev Immunol 2012 30 531 564 22224781 10.1146/annurev.immunol.25.022106.141623 PMC6066374

[B49] Povoleri GA Scotta C Nova-Lamperti EA John S Lombardi G Afzali B Thymic versus induced regulatory T cells – who regulates the regulators? Front Immunol 2013 4 169 23818888 10.3389/fimmu.2013.00169 PMC3694260

[B50] Cobb BS Hertweck A Smith J O’Connor E Graf D Cook T A role for Dicer in immune regulation J Exp Med 2006 203 2519 2527 17060477 10.1084/jem.20061692 PMC2118134

[B51] Zheng Y Josefowicz SZ Kas A Chu TT Gavin MA Rudensky AY Genome-wide analysis of FoxP3 target genes in developing and mature regulatory T cells Nature 2007 445 936 940 17237761 10.1038/nature05563

[B52] Lio CW Hsieh CS A two-step process for thymic regulatory T cell development Immunity 2008 28 100 111 18199417 10.1016/j.immuni.2007.11.021 PMC2248212

[B53] Burchill MA Yang J Vang KB Moon JJ Chu HH Lio CW Linked T cell receptor and cytokine signaling govern the development of the regulatory T cell repertoire Immunity 2008 28 112 121 18199418 10.1016/j.immuni.2007.11.022 PMC2430111

[B54] Vang KB Yang J Mahmud SA Burchill MA Vegoe AL Farrar MA IL-2, -7, and -15 but not stromal lymphopoetin, redundantly govern CD4 + FoxP3+ regulatory T cell development J Immunol 2008 181 3285 3290 18714000 10.4049/jimmunol.181.5.3285 PMC2810104

[B55] Lu LF Thai TH Calado D Chaudhry A Kubo M Tanaka K Foxp3-dependent microRNA155 confers competitive fitness to regulatory T cells by targeting SOCS1 protein Immunity 2009 30 80 91 19144316 10.1016/j.immuni.2008.11.010 PMC2654249

[B56] Lu LF Boldin MP Chaudhry A Lin LL Taganov KD Hanada T Function of miR-146a in controlling Treg cell-mediated regulation of Th1 responses Cell 2010 142 914 929 20850013 10.1016/j.cell.2010.08.012 PMC3049116

[B57] Rouas R Fayyad-Kazan H El Zein N Lewalle P Rothé F Simion A Human natural Treg microRNA signature: role of microRNA-31 and microRNA-21 in FOXP3 expression Eur J Immunol 2009 39 1608 1618 19408243 10.1002/eji.200838509

[B58] Liston A Lu LF O’Carroll D Tarakhovsky A Rudensky AY Dicer-dependent microRNA pathway safeguards regulatory T cell function J Exp Med 2008 205 1993 2004 18725526 10.1084/jem.20081062 PMC2526195

[B59] Zhou X Jeker LT Fife BT Thu S Anderson MS McManus MT Selective miRNA disruption in T reg cells leads to uncontrolled autoimmunity J Exp Med 2008 205 1983 1991 18725525 10.1084/jem.20080707 PMC2526194

[B60] Yao R Ma YL Liang W Li HH Ma ZJ Yu X MicroRNA-155 modulates Treg and Th17 cells differentiation and Th17 cell function by targeting SOCS1 PloS One 2012 7 e46082 23091595 10.1371/journal.pone.0046082 PMC3473054

[B61] Zhu J Yamane H Cote-Sierra J Guo L Paul WE GATA-3 promotes Th2 responses through three different mechanisms: induction of Th2 cytokine production, selective growth of Th2 cells and inhibition of Th1 cell-specific factors Cell Res 2006 16 3 10 16467870 10.1038/sj.cr.7310002

[B62] Rodriguez A Vigorito E Clare S Warren MV Couttet P Soond DR Requirement of bic/microRNA-155 for normal immune function Science 2007 316 608 611 17463290 10.1126/science.1139253 PMC2610435

[B63] Thai TH Calado DP Casola S Ansel KM Xiao C Xue Y Regulation of the germinal center response by microRNA-155 Science 2007 316 604 608 17463289 10.1126/science.1141229

[B64] Valadi H Ekstöm K Bossios A Sjöstrand M Lee JJ Lötvall JO Exosome-mediated transfer of mRNA and microRNAs is novel mechanism of genetic exchange between cells Nat Cell Biol 2007 9 654 659 17486113 10.1038/ncb1596

[B65] Liang H Huang L Cao J Zen K Chen X Zhang CY Regulation of mammalian gene expression by exogeneous microRNAs Wiley Interdiscip Rev RNA 2012 3 733 742 22740375 10.1002/wrna.1127

[B66] Chen X Liang H Zhang J Zen K Thang CY Secreted microRNAs: a new form of intercellular communication Trends Cell Biol 2012 22 125 132 22260888 10.1016/j.tcb.2011.12.001

[B67] Ludwig AK Giebel B Exosomes: small vesicles participating in intercellular communication Int J Biochem Cell Biol 2012 44 11 15 22024155 10.1016/j.biocel.2011.10.005

[B68] Ambros V The functions of animal microRNAs Nature 2004 431 350 355 15372042 10.1038/nature02871

[B69] Chen X Liang H Zhang J Zen K Zhang CY Horizontal transfer of microRNAs: molecular mechansism and clinical applications Protein Cell 2012 3 28 37 22314808 10.1007/s13238-012-2003-z PMC4875218

[B70] Raposo G Nijman HW Stoorvogel W Liejendecker R Harding CV Melief CJ B lymphocytes secrete antigen-presenting vesicles J Exp Med 1996 183 1161 1172 8642258 10.1084/jem.183.3.1161 PMC2192324

[B71] Mittelbrunn M Guitiérrez-Vásquez C Villarroya-Beltri C González S Sánchez-Cabo F González MA Unidirectional transfer of microRNA-loaded exosomes from T cells to antigen-presenting cells Nat Commun 2011 2 282 21505438 10.1038/ncomms1285 PMC3104548

[B72] Mittelbrunn M Sanchez-Madrid F Intercellular communication: diverse structures for exchange of genetic information Nat Rev Mol Cell Biol 2012 13 328 335 22510790 10.1038/nrm3335 PMC3738855

[B73] Gutiérrez-Vázquez C Villarroya-Beltri C Mittelbrunn M Sánchez-Madrid F Transfer of extracellular vesicles during immune cell-cell interactions Immunol Rev 2013 251 125 142 23278745 10.1111/imr.12013 PMC3740495

[B74] Skogberg G Gudmundsdottir J van der Post S Dandström K Bruhn S Benson M Characterization of human thymic exosomes PLoS One 2013 8 e67554 23844026 10.1371/journal.pone.0067554 PMC3699640

[B75] Melnik BC John SM Schmitz G Milk is not just food but most likely a genetic transfection system activating mTORC1 signaling for postnatal growth Nutr J 2013 12 103 23883112 10.1186/1475-2891-12-103 PMC3725179

[B76] Zhang L Hou D Chen X Li D Zhu L Zhang Y Exogenous plant MIR168a specifically targets mammalian LDLRAP1: evidence of cross-kingdom regulation by microRNA Cell Res 2012 22 107 126 21931358 10.1038/cr.2011.158 PMC3351925

[B77] Dickinson B Zhang Y Petrick JS Heck G Ivashuta S Marshall WS Lack of detectable oral bioavailability of plant microRNAs after feeding in mice Nat Biotechnol 2013 31 965 967 24213763 10.1038/nbt.2737

[B78] Weber JA Baxter DH Zhang S Huang DY Huang KH Lee MJ The microRNA spectrum in 12 body fluids Clin Chem 2010 56 1733 1741 20847327 10.1373/clinchem.2010.147405 PMC4846276

[B79] Hata T Murakami K Nakatani H Yamamoto Y Matsuda T Aoki N Isolation of bovine milk-derived microvesicles carrying mRNAs and microRNAs Biochem Biophys Res Commun 2010 396 528 533 20434431 10.1016/j.bbrc.2010.04.135

[B80] Chen X Gao C Li H Huang L Sun Q Dong Y Identification and characterization of microRNAs in raw milk during different periods of lactation, commercial fluid, and powdered milk products Cell Res 2010 20 1128 1137 20548333 10.1038/cr.2010.80

[B81] Izumi H Kosaka N Shimizu T Sekine K Ochiya T Takase M Bovine milk contains microRNA and messenger RNA that are stable under degradative conditions J Dairy Sci 2012 95 4831 4841 22916887 10.3168/jds.2012-5489

[B82] Admyre C Johansson SM Qazi KR Filén JJ Lahesmaa R Norman M Exosomes with immune modulatory features are present in human breast milk J Immunol 2007 179 1969 1978 17641064 10.4049/jimmunol.179.3.1969

[B83] Reinhardt TA Lippolis JD Nonnecke BJ Sacco RE Bovine milk exosome proteome J Proteomics 2012 75 1486 1492 22129587 10.1016/j.jprot.2011.11.017

[B84] Izumi H Kosaka N Shimizu T Sekine K Ochiya T Takase M Purification of RNA from milk whey Methods Mol Biol 2013 1024 191 201 23719952 10.1007/978-1-62703-453-1_15

[B85] Sun Q Chen X Yu J Zen K Zhang CY Li L Immune modulatory function of abundant immune-related microRNAs in microvesicles from bovine colostrum Protein Cell 2013 4 197 210 23483481 10.1007/s13238-013-2119-9 PMC4875502

[B86] Kosaka N Izumi H Sekine K Ochiya T microRNA as a new immune-regulatory agent in breast milk Silence 2010 1 7 20226005 10.1186/1758-907X-1-7 PMC2847997

[B87] Zhou Q Li M Wang X Li Q Wang T Zhu Q Immune-related microRNAs are abundant in breast milk exosomes Int J Biol Sci 2012 8 118 123 22211110 10.7150/ijbs.8.118 PMC3248653

[B88] van Niel G Raposo G Candalh C Boussac M Hershberg R Cerf-Benussan N Intestinal epithelial cells secrete exosome-like vesicles Gastroenterology 2001 121 337 349 11487543 10.1053/gast.2001.26263

[B89] Caby MP Lankar D Vincendeau-Scherrer C Raposo G Bonnerot C Exosomal-like vesicles are present in human blood plasma Int Immunol 2005 17 879 887 15908444 10.1093/intimm/dxh267

[B90] Corrado C Raimondo S Chiesi A Ciccia F De Leo G Alessandro R Exosomes as intercellular signaling organelles involved in health and disease: basic science and clinical applications Int J Mol Sci 2013 14 5338 5366 23466882 10.3390/ijms14035338 PMC3634447

[B91] Wang GJ Liu Y Qin A Shah SV Deng ZB Xiang X Thymus exosomes-like particles induce regulatory T cells J Immunol 2008 181 5242 5248 18832678 10.4049/jimmunol.181.8.5242 PMC4319673

[B92] Lässer C Alikhani VS Ekström K Eldh M Pareds PT Bossios A Human saliva, plasma and breast milk exosomes contain RNA: uptake by macrophages J Transl Med 2011 9 9 21235781 10.1186/1479-5876-9-9 PMC3033821

[B93] Boismenu R Rhein M Fischer WH Havran WL A role for CD81 in early T cell development Science 1996 271 198 200 8539618 10.1126/science.271.5246.198

[B94] Hulsmans M Holvoet P MicroRNA-containing microvesicles regulating inflammation in association with atherosclerotic disease Cardiovasc Res 2013 100 7 18 23774505 10.1093/cvr/cvt161

[B95] Olivieri F Spazzafumo L Santini GLazzarini R Albertini MC Rippo MR Age-related differences in the expression of circulating microRNAs: miR-21 a new circulating marker of inflammaging Mech Ageing Dev 2012 133 675 685 23041385 10.1016/j.mad.2012.09.004

[B96] Tam W Identification and characterization of human BIC, a gene on chromosome 21 that encodes a noncoding RNA Gene 2001 274 157 167 11675008 10.1016/s0378-1119(01)00612-6

[B97] Toker A Engelbert D Garg G Polansky JK Floess S Miyao T Active demethylation of the Foxp3 locus leads to the generation of stable regulatory T cells within the thymus J Immunol 2013 190 3180 3188 23420886 10.4049/jimmunol.1203473

[B98] Polansky JK Schreiber L Thelemann C Ludwig L Krüger M Baumgrass R Methylation matters: binding of Ets-1 to the demethylated Foxp3 gene contributes to the stabilization of FoxP3 expression in regulatory T cells J Mol Med (Berl) 2010 88 1029 1040 20574810 10.1007/s00109-010-0642-1 PMC2943068

[B99] Hinz D Bauer M Röder S Olek S Huehn J Sack U LINA study group Cord blood Tregs with stable FOXP3 expression are influenced by prenatal environment and associated with atopic dermatitis at the age of one year Allergy 2012 67 380 389 22187950 10.1111/j.1398-9995.2011.02767.x

[B100] Koh KP Rao A DNA methylation and methylcytosine oxidation in cell fate decisions Curr Opin Cell Biol 2013 25 152 161 23498662 10.1016/j.ceb.2013.02.014 PMC3649866

[B101] Ko M Bandukwala HS Chavez L Aijö T Pastor WA Segal MF Modulation of TET2 expression and 5-methylcytosine oxidation by the CXXC domain protein IDAX Nature 2013 497 122 126 23563267 10.1038/nature12052 PMC3643997

[B102] Dunican DD Pennings S Meeha RR The CXXC-TET bridge–mind the methylation gap! Cell Res 2013 23 973 974 23711677 10.1038/cr.2013.71 PMC3731560

[B103] Lal G Bromberg JS Epigenetic mechanisms of regulation of Foxp3 expression Blood 2009 114 3727 3735 19641188 10.1182/blood-2009-05-219584 PMC2773485

[B104] Cheng J Guo S Chen S Mastriano SJ Liu C D’Alessio AC An extensive network of TET2-targeting microRNAs regulates malignant hematopoiesis Cell Rep 2013 5 471 481 24120864 10.1016/j.celrep.2013.08.050 PMC3834864

[B105] Pan W Zhu S Yuan M Cui H Wang L Luo X MicroRNA-21 and microRNA-148a contribute to DNA hypomethylation in Lupus CD4+ T cells by directly and indirectly targeting DNA methyltransferase 1 J Immunol 2010 184 6773 6781 20483747 10.4049/jimmunol.0904060

[B106] Qin H Zhu X Liang J Wu J Yang Y Wang S MicroRNA-29b contributes to DNA hypomethylation of CD4+ T cells in systemic lupus erythematodes by indirectly targeting DNA methyltransferase 1 J Dermatol Sci 2013 69 61 67 23142053 10.1016/j.jdermsci.2012.10.011

[B107] Ho IC Hodge MR Rooney JW Glimcher LH The proto-oncogene c-maf is responsible for tissue-specific expression of interleukin-4 Cell 1996 85 973 983 8674125 10.1016/s0092-8674(00)81299-4

[B108] Kim JI Ho IC Grusby MJ Glimcher LH The transcription factor c-Maf controls the production of interleukin-4 but not other Th2 cytokines Immunity 1999 10 745 751 10403649 10.1016/s1074-7613(00)80073-4

[B109] Vigorito E Kohlhaas S Lu D Leyland R miR-155: an ancient regulator of the immune system Immunol Rev 2013 253 146 157 23550644 10.1111/imr.12057

[B110] Corey SJ Mehta HM Stein PL Two SHIPs passing in the middle of the immune system Eur J Immunol 2012 42 1681 1684 22696261 10.1002/eji.201242706

[B111] Srivastava N Sudan R Kerr WG Role of inositol poly-phosphatases and their targets in T cell biology Front Immunol 2013 4 288 24069021 10.3389/fimmu.2013.00288 PMC3779868

[B112] Collazo MM Paraiso KH Park MY Hazen AL Kerr WG Lineage extrinsic and intrinsic control of immunoregulatory cell numbers by SHIP Eur J Immunol 2012 42 1785 1795 22535653 10.1002/eji.201142092 PMC3816569

[B113] O’Connell RM Chaudhuri AA Rao DS Baltimore D Inositol phosphatase SHIP1 is a primary target of miR-155 Proc Natl Acad Sci U S A 2009 106 7113 7118 19359473 10.1073/pnas.0902636106 PMC2678424

[B114] Vigorioto E Perks KL Abreu-Goodger C Bunting S Xiang Z Kohlhaas S microRNA-155 regulates the generation of immunoglobulin class-switched plasma cells Immunity 2007 27 847 859 18055230 10.1016/j.immuni.2007.10.009 PMC4135426

[B115] Stütz AM Woisetschläger M Functional synergism of STAT6 with either NF-kappaB or PU.1 to mediate IL-4-induced activation of the IgE germline gene transcription J Immunol 1999 163 4383 4391 10510379

[B116] Stütz AM Hoeck J Natt F Cuenoud B Woisetschläger M Inhibition of interleukin-4- and CD40-induced IgE germline gene promoter activity by 2′-aminoethoxy-modified triplex-forming oligonucleotides J Biol Chem 2001 276 11759 11765 11278649 10.1074/jbc.M010260200

[B117] Gauchat JF Lebman DA Coffman RL Gascan H de Vries JE Stucture and expression of germline epsilon transcripts in human B cells induced by interleukin 4 to switch to IgE production J Exp Med 1990 172 463 473 1695667 10.1084/jem.172.2.463 PMC2188335

[B118] Nishiyama C Molecular mechanism of allergy-related gene regulation and hematopoietic cell development by transcription factors Biosci Biotechnol Biochem 2006 70 1 9 16428815 10.1271/bbb.70.1

[B119] Choi SY Sohn MH Kwon BC Kim KE CTLA-4 expression in T cell of patients with atopic dermatitis Pediatr Allergy Immunol 2005 16 422 427 16101935 10.1111/j.1399-3038.2005.00274.x

[B120] Sonkoly E Janson P Majuri ML Savinko T Fyhrquist N Eidsmo L MiR-155 is overexpressed in patients with atopic dermatitis and modulates T-cell proliferative responses by targeting cytotoxic T lymphocyte-associated antigen 4 J Allergy Clin Immunol 2010 126 581 589 20673989 10.1016/j.jaci.2010.05.045

[B121] Wing K Onishi Y Prieto-Martin P Yamaguchi T Miyara M Fehervari Z CTLA-4 control over Foxp3+ regulatory T cell function Science 2008 322 271 275 18845758 10.1126/science.1160062

[B122] Prescott SL Effects of early cigarette smoke exposure on early immune development and respiratory diseases Paediatr Respir Rev 2008 9 3 10 18280974 10.1016/j.prrv.2007.11.004

[B123] Lauener R Birchler T Adamski J Braun-Fahrländer C Bufe A Herz U Expression of CD14 and Toll-like receptor 2 in farmers and non-farmers’ children Lancet 2002 360 465 466 12241724 10.1016/S0140-6736(02)09641-1

[B124] Ege MJ Bieli C Frei R van Strien RT Riedler J Ublagger E Prenatal farm exposure is related to the expression of receptors of the innate immunity and to atopic sensitization in schoo-age children J Allergy Clin Immunol 2006 117 817 823 16630939 10.1016/j.jaci.2005.12.1307

[B125] O’Neill LA Sheedy FJ McCoy CE MicroRNAs: the fine-tuners of Toll-like receptor signalling Nat Rev Immunol 2011 11 163 175 21331081 10.1038/nri2957

[B126] Nahid MA Satoh M Chan EK MicroRNA in TLR signaling and endotoxin tolerance Cell Mol Immunol 2011 8 388 403 21822296 10.1038/cmi.2011.26 PMC3618661

[B127] O’Connell RM Taganov KD Boldin MP Chneg G Baltimore D MicroRNA-155 is induced during the macrophage inflammatory response Proc Natl Acad Sci U S A 2007 104 1604 1609 17242365 10.1073/pnas.0610731104 PMC1780072

[B128] Tili E Michaille JJ Cimino A Costinean S Dumitru CD Adair B Modulation of miR-155 and miR-125b levels following lipopolysaccharide/TNF-alpha stimulation and their possible roles in regulating the response to endotoxin shock J Immunol 2007 179 5082 5089 17911593 10.4049/jimmunol.179.8.5082

[B129] McCoy CE Sheedy FJ Qualls JE Doyle SL Quinn SR Murray PJ IL-10 inhibits miR-155 induction by toll-like receptors J Biol Chem 2010 285 20492 20498 20435894 10.1074/jbc.M110.102111 PMC2898307

[B130] Taganov KD Boldin MP Chang KJ Baltimore D NF-kappaB-dependent induction of microRNA miR-146, an inhibitor targeted to signaling proteins of innate immune responses Proc Natl Acad Sci U S A 2006 103 12481 12486 16885212 10.1073/pnas.0605298103 PMC1567904

[B131] Sheedy FJ Palsson-McDermott E Hennessy EJ Martin C O’Leary JJ Ruan Q Negative regulation of TLR4 via targeting of the proinflammatory tumor suppressor PDCD4 by the microRNA miR-21 Nat Immunol 2010 11 141 147 19946272 10.1038/ni.1828

[B132] Worm J Stenvang J Petri A Frederiksen KS Obad S Elmén J Silencing of microRNA-155 in mice during acute inflammatory response leads to derepression of c/ebp beta and down-regulation of G-CSF Nucleic Acid Res 2009 37 5784 5792 19596814 10.1093/nar/gkp577 PMC2761263

[B133] Ceppi M Peireira PM Dunand-Sauthier I Barras E Reith W Santos MA MicroRNA-155 modulates the interleukin-1 signaling pathway in activated human monocyte-derived dendritic cells Proc Natl Acad Sci U S A 2009 106 2735 2740 19193853 10.1073/pnas.0811073106 PMC2650335

[B134] Singh PP Smith VL Karakousis PC Schorey JS Exosomes isolated from mycobacteria-infected mice or cultured macrophages can recruit and activate immune cells in vitro and in vivo J Immunol 2012 189 777 785 22723519 10.4049/jimmunol.1103638 PMC3685416

[B135] Delorme-Axford E Donker RB Mouillet JF Chu T Bayer A Quyang Y Human placental trophoblasts confer viral resistance to recipient cells Proc Natl Acad Sci U S A 2013 110 12048 12053 23818581 10.1073/pnas.1304718110 PMC3718097

[B136] Dai Y Diao Z Sun H Li R Qiu Z Hu Y MicroRNA-155 is involved in the remodeling of the human-trophoblast-derived HTR-8/SVneo cells induced by lipopolysaccharides Hum Reprod 2011 26 1882 1891 21515911 10.1093/humrep/der118

[B137] Bullerdiek J Flor I Exosome-delivered microRNAs of “chromosome 19 microRNA cluster” as immunomodulators in pregnancy and tumorigenesis Mol Cytogenet 2012 5 27 22559272 10.1186/1755-8166-5-27 PMC3388007

[B138] Schaub B Liu J Höppler S Schleich I Huehn J Olek S Maternal farm exposure modulates neonatal immune mechansims through regulatory T cells J Allergy Clin Immunol 2009 123 774 782 19348917 10.1016/j.jaci.2009.01.056

[B139] Nakagawa R Naka T Tsutsui H Fujimotot M Kimura A Abe T SOCS-1 participates in negative regulation of LPS responses Immunity 2002 17 677 687 12433373 10.1016/s1074-7613(02)00449-1

[B140] Kinjyo I Hanada T Inagaki-Ohara K Mori H Aki D Ohishi M SOCS1/JAB is a negative regulator of LPS-induced macrophage activation Immunity 2002 17 583 591 12433365 10.1016/s1074-7613(02)00446-6

[B141] Munch EM Harris AA Mohammad M Benham AL Pejerrey SM Showalter L Transcriptome profiling of microRNA by next-gen deep sequencing reveals known and novel species in the lipid fraction of human breast milk PLoS One 2013 8 e50564 23418415 10.1371/journal.pone.0050564 PMC3572105

[B142] Ip S Chung M Raman G Chew P Magula N De Vine D Breastfeeding and maternal and infant health outcomes in developed countries Evid Rep Technol Assess (Full Rep) 2007 153 1 186 17764214 PMC4781366

[B143] Diepgen TL Blettner M Analysis of familial aggregation of atopic eczema and other diseases by odds ratio regression models J Invest Dermatol 1996 106 977 981 8618061 10.1111/1523-1747.ep12338475

[B144] Dold S Wjst M von Mutius E Reitmeir P Stiepel E Genetic risk for asthma, allergic rhinitis, and atopic dermatitis Arch Dis Child 1992 67 1018 1022 1520004 10.1136/adc.67.8.1018 PMC1793604

[B145] Ruiz RG Kemeny DM Price JF Higher risk of infantile atopic dermatitis from maternal atopy than from paternal atopy Clin Exp Allergy 1992 22 762 766 1525695 10.1111/j.1365-2222.1992.tb02816.x

[B146] Greer FR Sicherer SH Burks W American Academy of Pediatrics Committee on Nutrition and Section on Allergy and Immunology Effects of early nutritional interventions on the development of atopic disease in infants and children: the role of maternal dietary restriction, breastfeeding, timing of introduction of complementary foods, and hydrolyzed formulas Pediatrics 2008 121 183 191 18166574 10.1542/peds.2007-3022

[B147] Lee SY Kang MJ Kwon JW Parks KS Hong SJ Breast feeding might have protective effects on atopy in children with the CD14C-159CT/CC genotype Allergy Asthma Immunol Res 2013 5 239 241 23814678 10.4168/aair.2013.5.4.239 PMC3695239

[B148] Simpson RJ Lim JW Moritz RL Mathivanan S Exosomes: proteomic insights and diagnostic potential Expert Rev Proteomics 2009 6 267 283 19489699 10.1586/epr.09.17

[B149] Choi DS Kim DK Kim YK Gho YS Proteomics, transcriptomics and lipidomics of exosomes and ectosomes Proteomics 2013 13 1554 1571 23401200 10.1002/pmic.201200329

[B150] Kosaka N Yoshioka Y Hagiwara K Tominaga N Katsuda T Ochiya T Trash or treasure: extracellular microRNAs and cell-to-cell communication Front Genet 2013 4 173 24046777 10.3389/fgene.2013.00173 PMC3763217

[B151] Smyth LM Ratnasothy K Tsang JY Boardman D Warley A Lechler R CD73 expression on extracellular vesicles derived from CD4 + CD25 + FoxP3+ T cells contributes to their regulatory function Eur J Immunol 2013 43 2430 2440 23749427 10.1002/eji.201242909

